# Aptameric Recognition-Modulated Electroactivity of Poly(4-Styrenesolfonic Acid)-Doped Polyaniline Films for Single-Shot Detection of Tetrodotoxin

**DOI:** 10.3390/s150922547

**Published:** 2015-09-08

**Authors:** Gertrude Fomo, Tesfaye T. Waryo, Christopher E. Sunday, Abd A. Baleg, Priscilla G. Baker, Emmanuel I. Iwuoha

**Affiliations:** SensorLab, Chemistry Department, University of the Western Cape, P. Bag X17, Bellville, Cape Town 7535, South Africa, E-Mails: 3262807@myuwc.ac.za (G.F.); twaryo@uwc.ac.za (T.T.W.); csunday@uwc.ac.za (C.E.S.); abaleg@uwc.ac.za (A.A.B.); pbaker@uwc.ac.za (P.G.B.)

**Keywords:** tetrodotoxin sensor, aptamer, polyaniline, puffer fish

## Abstract

The work being reported is the first electrochemical sensor for tetrodotoxin (TTX). It was developed on a glassy carbon electrodes (C) that was modified with poly(4-styrenesolfonic acid)-doped polyaniline film (PANI/PSSA). An amine-end functionalized TTX-binding aptamer, *5*′*-NH_2_-AAAAATTTCACACGGGTGCCTCGGCTGTCC-3*′ (NH_2_-Apt), was grafted via covalent glutaraldehyde (glu) cross-linking. The resulting aptasensor (C//PANI^+^/PSSA-glu-NH_2-_Apt) was interrogated by cyclic voltammetry (CV) and electrochemical impedance spectroscopy (EIS) in sodium acetate buffer (NaOAc, pH 4.8) before and after 30 min incubation in standard TTX solutions. Both CV and EIS results confirmed that the binding of the analyte to the immobilized aptamer modulated the electrochemical properties of the sensor: particularly the charge transfer resistance (*R*_ct_) of the PANI^+^/PSSA film, which served as a signal reporter. Based on the *R*_ct_ calibration curve of the TTX aptasensor, the values of the dynamic linear range (DLR), sensitivity and limit of detection (LOD) of the sensor were determined to be 0.23–1.07 ng·mL^−1^ TTX, 134.88 ± 11.42 Ω·ng·mL^−1^ and 0.199 ng·mL^−1^, respectively. Further studies are being planned to improve the DLR as well as to evaluate selectivity and matrix effects in real samples.

## 1. Introduction

Tetrodotoxin (TTX), known as puffer fish toxin, is one of the most potent nonpeptidic neurotoxins because of its frequent involvement in fatal food poisoning, its unique chemical structure, and its specific action of blocking sodium channels of excitable membranes [1]. Through the study of cultured puffer fish, it was discovered that tetrodotoxin was not metabolically produced within the fish. Instead, it is synthesized by several bacterial species, including strains of the families *vibrionaceae* and *pseudomonas* [2]. Several approaches for the detection of tetrodotoxin identified using the enzyme-linked immunosorbent assay (ELISA) technique were reported by Neagu and co-workers and involved the use of the tetrodotoxin with alkaline phosphatase (AP) [3]. On the other hand, high performance liquid chromatography (HPLC) using fluorescent detection following post-column alkaline degradation and a sample preparation procedure for the analysis were established to quantitatively detect tetrodotoxin in gastropods and puffer fishes [4]. Recently, Taylor *et al.* and Yakes *et al.* reported a quantitative antibody-based detection of tetrodotoxin by inhibition assay with a surface plasmon resonance (SPR) sensor and the result was compared to the analytical methods [5,6]. It is well known that these above-mentioned analytical methods have some limitations compared to electrochemical methods [7]. A biosensor is an analytical device incorporating a biorecognition element intimately associated with or integrated within a transducer that converts the physicochemical information into an electrical signal. Biosensor devices are in principle adaptable, simple to prepare, selective and specific, accurate, and timely, with minimal sample pre-treatment involved [8]. To the best of our knowledge, no biosensor based on aptamer has been reported for the detection of tetrodotoxin.

Aptamers are short oligonucleotides (DNA/RNA) that can bind with high affinity and specificity to a wide range of target molecules, such as drugs, proteins, toxins or other organic and inorganic molecules [9,10].Due to their easy and quick preparation, cost-effectiveness, small size and versatility, aptamers have become useful tools for the validation of intracellular and extracellular targets [11,12,13]. A continuously growing number of nucleic acid aptamers are used as research tools to study specific protein functions and interactions [14,15]. Many reports on the aptamer-based biosensors for detection of various proteins [16,17,18] and toxin [19,20,21,22] have been investigated. One important parameter in the fabrication of aptamer-based biosensors is the method for attachment of the aptamer. As well as the immobilization of enzyme, peptides, ligands or other biomolecules, aptamer have the same method for immobilization since there is no general universally applicable method of particular molecule immobilization [23]. However, three principal methods can be used for immobilization of aptamer such as adsorption, covalence and the cross-linking method. For this study, the covalent binding method, which is the reaction involving the formation of covalent bonds between the functional groups belonging to the biomolecule (aptamer) and the support matrix (conducting polyaniline), was applied. Conducting electroactive polyanilines have received considerable attention from both academia and industry because of their many potential applications such as artificial muscles and sensors [24]. The application of conducting polymers to electrochemical biosensors is mainly based on the idea that they can improve direct electron transport between the biomolecule and electrode surface in amperometric biosensors [25]. In this context, ordered monolayers of conducting p-doped PANI/PSSA films prepared using electrochemical polymerization [26] can perhaps be used.

This paper aimed to carry out the first electrochemical analysis of tetrodotoxin involving affinity interactions, by using glassy carbon electrode modified with selective aptamer immobilized on p-doped PANI/PSSA electroactive polymer platforms. The aptamer functioned as the biorecognition probe for the amperometric and impedimetric determination of TTX.

## 2. Experimental Section

### 2.1. Chemicals and Materials

Tetrodotoxin (96%) was purchased from Latoxan (Valence, France). Phosphoric acid (H_3_PO_3_) (85 wt%) purchased from Sigma-Aldrich (St. Louis, MO, USA). Aniline, glutaraldehyde (≥50 wt%), poly(4-styrenesulfonic acid) (18 wt% in water), sodium acetate anhydrous (99%), acetic acid, and glacial (99.7%), were procured from Sigma-Aldrich. The TTX aptamer used in the present study was the aminylated DNA-aptamer sequence, *5′-NH_2_-AAAAATTTCACACGGGTGCCTCGGCTGTCC-3′* (NH_2_-Apt), chosen on the basis of the original work of *Shao et al.* [27] and custom-produced at Inqaba Biotec (Pretoria, South Africa).

### 2.2. Electrochemical Set-Up and Measurements

A conventional three-electrode electrochemical cell was used for electrochemical studies. The electrolyte (0.1 M sodium acetate buffer (NaOAc, pH 4.8) was always degassed and then blanketed over with argon gas during experiments. A modified glassy carbon working electrode (WE, d = 3.0 mm, BAS Inc., West Lafayette, IN, USA), a Ag/AgCl reference electrode (BAS Inc., West Lafayette, IN, USA) and a platinum wire (99.9%, Sigma-Aldrich) counter electrode were employed. The surface of the working electrode was cleaned by successively polishing with alumina polishing powders (Buehler, Lake Bluff, IL, USA) of 1 µm, 0.3 µm and 0.05 µm particle sizes in that order. After the polishing step, the electrode was rinsed with ultra-pure water and then placed in an ultrasonic bath containing absolute ethanol and sonicated for 10 min and rinsed with ultra-pure water.

### 2.3. Preparation of TTX Aptasensor

#### 2.3.1. Electrodeposition of Polystyrene Sulfonic Acid-Doped Polyaniline Film (PSSA/PANI)

The PANI/PSSA film was first electrochemically deposited on a bare glassy carbon electrode (C) from an aqueous solution of H_3_PO_3_ (0.1 M) containing 0.05 M aniline (monomer) and 0.025 M PSSA, by potentiodynamic electro-oxidation of the monomer at a scan rate of 50 mV·s^−1^ from -0.1 V to +1.3 V for five cycles. The resulting p-doped PANI/PSSA- or pernigraniline blue/PSSA-coated electrode, herein referred to as PSSA/PANI//C, was immediately rinsed with ethanol and deionized water in order to remove excess reactants and soluble intermediates.

#### 2.3.2. Immobilization of the Aminylated Anti-TTX Aptamer (Apt-NH_2_) onto PANI/PSSA Film

**Figure 1:**
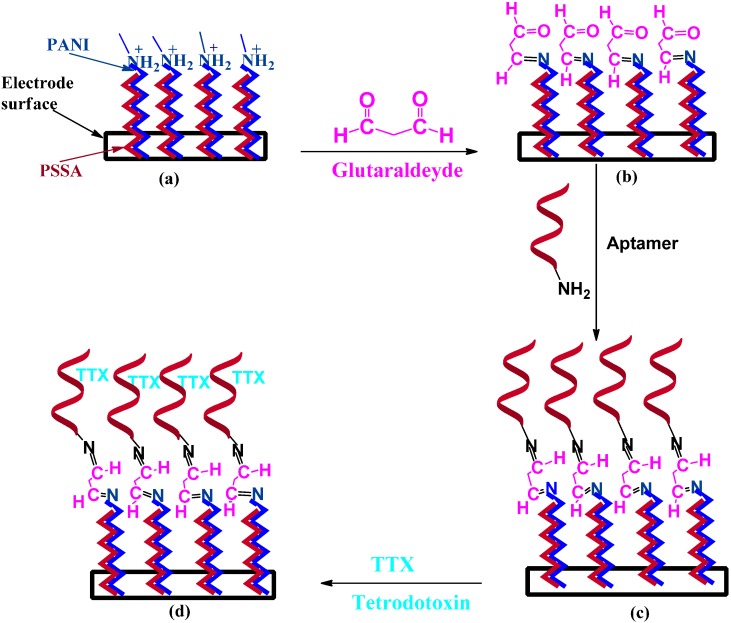
Schematics for the development of the TTX aptasensor: (**a**) electrodeposition of PSSA-doped polyaniline onto the carbon electrode; (**b**) glutaraldehyde-functionalization of PANI/PSSA film; (**c**) immobilization of NH_2_-aptamer on glu-PANI/PSSA/GC; and (**d**) TTX detection.

Immobilization of Apt-NH_2_ onto the PANI/PSSA layer was effected by using glutaraldehyde as a cross-linker, which was based on a procedure reported in the literature [28,29]. Glutaraldehyde is a di-aldehyde which forms a covalent bond between its aldehyde group and an amine group of the binding molecule [28]. In the present case, –CHO functional groups of glutaraldehyde reacted with the amine group of PANI chains at one end and the amine group of aminylated DNA aptamer at the other, resulting in the formation of stable covalent bonds [28]. In a typical procedure the PANI/PSSA//C surface was reacted with aqueous 2% glutaraldehyde solution for 4 h at room temperature (25 °C). The resulting glutaraldehyde-functionalized film (glu-PANI/PSSA) was then rinsed with de-ionized water and further exposed to a solution of Apt-NH_2_ (2 µM) in acetate buffer (0.1 M) for about 4 h at 4 °C. The aptasensor produced (Apt-NH_2_-glu-PANI/PSSA//C) was rinsed with aliquots of 0.1 M acetate buffer and stored at 4 °C when not in use. Figure 1 is a schematic representation of the preparation of the Apt-NH_2_-glu-PANI/PSSA//C aptasensor.

### 2.4. Microscopic and Spectroscopic Characterization

The UV-Vis absorption characteristics of the Apt-NH_2_-glu-PANI/PSSA film was studied with a Nicolet Evolution 100 Spectrometer (Thermo Fisher Scientific, Waltham, MA, USA). The scanning electron microscopy (SEM) imaging of the film was performed with a Carl Zeiss Auriga HRSEM (Carl Zeiss Microscopy GmbH, Oberkochen). For the UV/Vis analysisthe film was dispersed in dimethyl formamide (DMF) under sonication for about 20–30 min. For SEM imaging purposes, carbon screen printed carbon electrodes (C-SPE) obtained from DropSens (Llanera, Asturias, Spain) were used as platforms.

### 2.5. Electrochemical Studies

Cyclic voltammetry (CV) and electrochemical impedance spectroscopy (EIS) were performed with an IM6ex ZAHNER Elektrik Electrochemical Workstation (ZAHNER-Elektrik GmbH & Co. KG, Kronach, Germany) in 0.1 M NaOAc, pH 4.8 and (at a frequency range of 100 kHz to 100 mHz for EIS measurements). The aptasensor was incubated in TTX solutions for about 30 min at room temperature. After incubation of the sensor in TTX the aptasensor surface was rinsed with the NaOAc buffer to remove loosely held TTX molecules before making measurements.

## 3. Results and Discussion

### 3.1. SEM and UV-Vis Analysis

**Figure 2:**
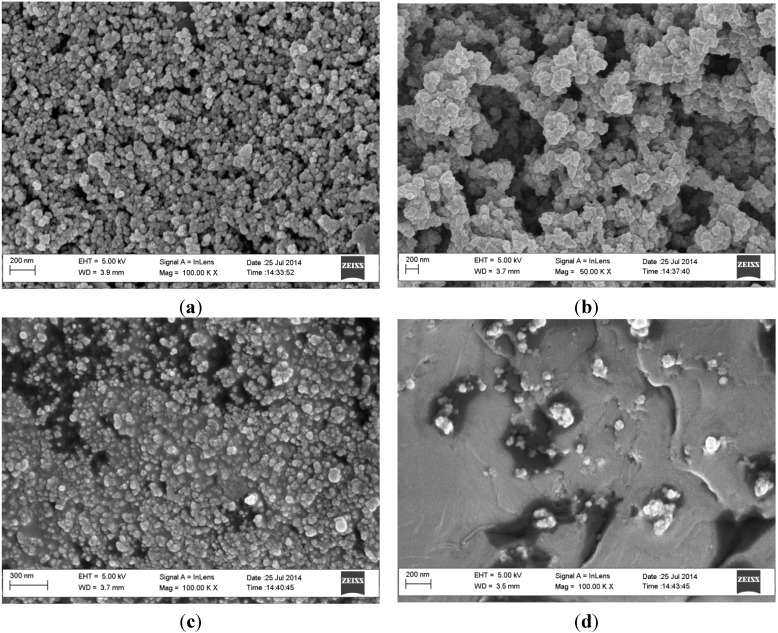
Scanning electron micrographs of (**a**) bare C-SPE; (**b**) PANI/PSSA//C-SPE; (**c**) Apt-NH_2_-glu-PANI/PSSA//C-SPE; and (**d**) TTX-Apt-NH_2_-glu-PANI/PSSA//C-SPE (where C-SPE is carbon screen printed electrode).

Figure 2a–d shows the SEM images obtained at different stages of the preparation of the aptasensor on a screen printed carbon electrode (C-SPE). According to Figure 2b, p-doped PANI/PSSA films form clusters of flake-shaped particles with diameters of 70–100 nm, which is in contrast to the more or less spherically shaped and uniformly dispersed 25–50 nm diameter carbon particles of the C-SPE platform (Figure 2a). The PANI/PSSA particles appear to have grown only over certain sites on the C-SPE, which can be understood as being because of the fact that not all of its carbon particles are accessible for electrochemical reactions.

Upon the exposure of the PANI/PSSA film to glutaraldehyde, followed by NH_2_-aptamer, new cloudy particle shapes appeared (Figure 2c), confirming that the aptamer molecules were successfully attached onto the PANI/PSSA surface via the glutaraldehyde cross-linking process. It also shows that the PANI/PSSA framework reorganized itself into clusters of smaller spherical particles with diameter of 22 to 44 nm on formation of the Apt-NH_2_-glu-PANI/PSSA supra-molecular assembly, completing the aptasensor fabrication process. The SEM image underwent an even more morphological change after exposure of the aptasensor to the TTX solution, as can be seen in Figure 2d. Of the three major types of surface features in this image (Figure 2d), the dark regions and the random clusters of particles were identified as left-over of non-TTX-binding areas, while the ambient smooth and gray shaded region are where agglutination occurred between the aptamer and TTX. The darkest regions should definitely represent C-SPE sites without PANI/PSSA and hence without aptamer. This indicates that there is a strong binding affinity between the immobilized aptamer and TTX and that the preparation of the aptasensor was successful.

**Figure 3:**
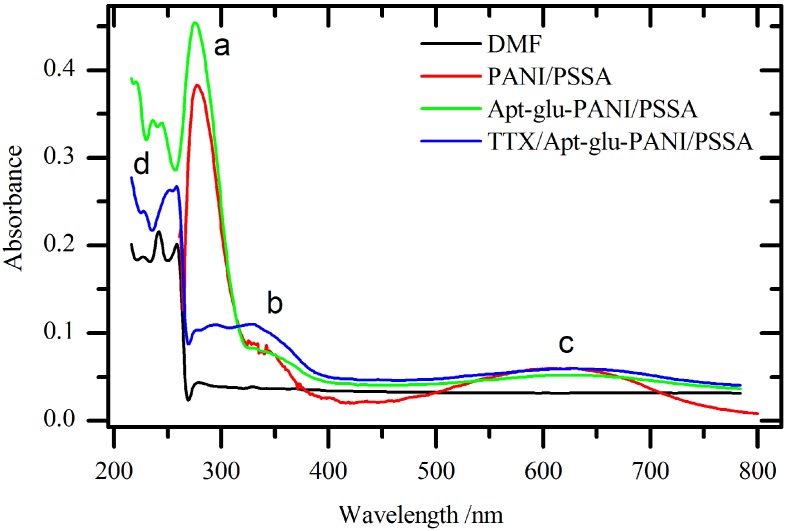
UV-Vis spectra of PANI/PSSA, Apt-NH_2_-glu-PANI/PSSA, and TTX-Apt-NH_2_-glu-PANI/PSSA composite materials dispersed in DMF.

Figure 3 displays the UV-Vis spectra of PANI/PSSA, Apt-NH_2_-glu-PANI/PSSA, and TTX-Apt-NH_2_-glu-PANI/PSSA that were dispersed or dissolved in dimethyl formamide (DMF). Figure 3 shows that in each step of the aptasensor formation four characteristic absorptions bands (a, b, c, and d) were observed. The (d) absorption band can be attributed to chromophores present in DMF. The absorption band between 275.5 and 294.9 nm is due to the π → π* transition of benzenoid ring of PANI and PSSA. The absorption band (a) corresponds to the C=C chromophores with π → π* electronic transition, and the absorbance of this band increases when Apt-NH_2_-glu-PANI/PSSA is formed, which is due to the increase of concentration of C=C in the aptamer system. A huge difference was observed for absorption band (a) when the aptasensor was exposed TTX (TTX-Apt-NH_2_-glu-PANI/PSSA). The 88% decrease in the absorbance of band (a) when the aptasensor binds TTX may be due to the delocalization of electrons within composite polymer chain. This delocalization makes the C=C chromophores less stable or unavailable due to the formation of hydrogen bonds between O and N atoms of the aptamer and the H atom of TTX. The absorption band at 327.4 nm (b) corresponds to n → σ* transition of the N−C chromophores of PANI. When the TTX is attached to the aptasensor, the absorption (b) becomes more pronounced, which is indicative of the reactivity of the binding of TTX to the aptamer. No significant change was observed on the broad band at 623.9 nm (c) for each of the three composite polymer systems. The band corresponds to the shift of electron from the benzenoid to the quinoid rings of PANI due to π → π* electronic transition.

### 3.2. Electroanalysis of Aptasensor

**Figure 4:**
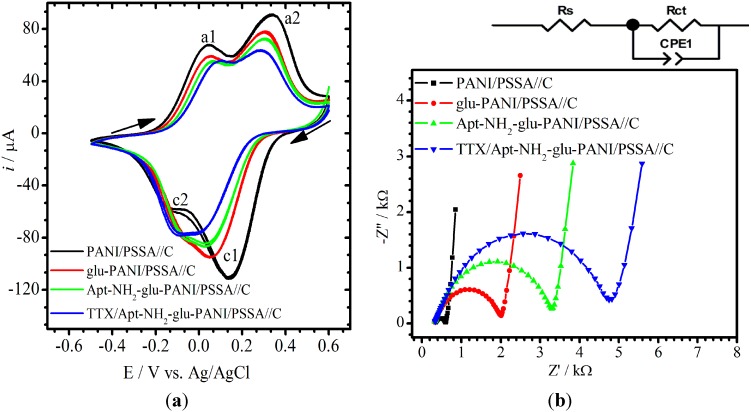
CVs (**a**) and EIS spectra (**b**) of TTX aptasensor and its electrode materials. Conditions: NaOAc buffer (0.1 M and pH 4.8); initial CV scan direction is cathodic; and EIS’s *E* = 0.010 V and *f* = 100 mHz–100 kHz. The equivalent circuit on top of (**b**) was used to fit the Nyquist plots (*R*_s_, *R*_ct_ and *CPE1* are solution resistance, charge transfer resistance and constant phase element, respectively).

Figure 4a shows an overlay of the CVs of PANI/PSSA//C (black), glu-PANI/PSSA//C (red), Apt-NH_2_-glu-PANI/PSSA//C (green) and TTX-Apt-NH_2_-glu-PANI/PSSA (blue: obtained after 30 min exposure of the aptasensor to standard solution of TTX (5 μM)). The EIS spectra (Nyquist plots) in Figure 4b were obtained with the same cell solution used for CV by setting the dc bias potentials to the formal potentials of a1/c1 and a2/c1 redox couples. The a1/c1 and a2/c1, regardless of the film-composition, are due to the PANI film exhibiting two consecutive electrode reactions as already well described in the literature [30,31]. In this study, since the PANI was made to be deposited in its fully oxidized form (or pernigraniline salt) and always interrogated with cathodic initial scans, c1 is the first observed cathodic peak, followed by the second cathodic peak c2, whereas peaks a2 and a1 are the respective anodic reverse peaks. In brief, while c1 represents a two-electron reduction of bipolaronic segments in pernigraniline-PANI into polarons to form the emeraldine PANI, c2 is the reduction of the latter to neutral leucoemeraldine PANI via another two-electron process. The decrease of the peak current around −0.1 V and 0.01 V demonstrates an effective affinity binding detection of TTX on the Apt-NH_2_-glu-PANI/PSSA//C electrode. The cathodic peaks (emeraldine and neutral leucoemeraldine PANI formation peaks) decrease as the PANI-PSSA electrode loses it conductivity due to modification with glutaraldehyde, aptamer and TTX [32].

By following the changes in CV characteristics through the progress of sensor fabrication, one could observe that the peak currents decreased when glutaraldehyde was attached to the PANI/PSSA surface. Further decreases were again observed after the Apt-NH_2_ molecules were attached to the glu-PANI/PSSA surface and then also after the resulting aptasensor was exposed to the TTX solution. At same time, the formal potentials, approximated as average of the peak pairs, also shifted to lower and lower values at each successive step of the aptasensor fabrication, particularly for the c1/a2 redox peak pairs. A possible explanation for the above effects on peak currents and potentials would be that the microenvironment of PANI’s electron transfer reactions was successively altered as the different molecular additives and functional groups were grafted into it. For the EIS measurements, the redox system of the c2/a1 peak pairs was chosen because its charge transfer resistance (*R*_ct_) was found to vary more favorably and more significantly at each stage of the fabrication process as well as after binding of TTX in contrast to the c1/a2 redox system. For analytical signal collection purposes, the simplified Randles’ cell circuit shown in Figure 4b (top) was used to fit (χ^2^ was in the order of 10^−4^) the semi-circle or ZARC segments of the EIS spectra of PANI/PSSA//C (black), glu-PANI/PSSA//C (red), Apt-NH_2_-glu-PANI/PSSA//C and TTX/Apt-NH_2_-glu-PANI/PSSA//C (blue).

The *R*_ct_ increased after the immobilization of glutaraldehyde due to its non-conducting behavior [29,33]. The further increase in *R*_ct_ following the immobilization of Apt-NH_2_ might be attributed to the negatively charged backbone of aptamer causing repulsion of charge balancing counter ions [29], in addition to its steric and insulating effects. This is in agreement with the trend observed in the CV studies, which is not unexpected considering the fact that the current should be inversely related to the *R*_ct_ [34,35].

### 3.3. Optimization of the Aptasensor

Experiments were performed to optimize the concentrations of the cross-linker (glutaraldehyde) and recognition agent (aptamer) required for the development of the TTX aptasensor. The percentage change in *R*_ct_ $\left(\%\Delta R_{ct}=100\times (R_{ct,\text{​}after}-R_{ct,\text{​}before})/R_{ct,\text{​}before}\right)$ before and after exposure to TTX solution was plotted in order to find the critical concentrations of these reagents required in the sensor. The %Δ*R*_ct_ increased with increasing concentration of glutaraldehyde in the range 0–0.05 mM, but decreased in the range 0.05–2 mM. With regard to the aptamer the %Δ*R*_ct_ increased with increasing concentration of aptamer in the range 0–0.05 µM and decreased in the 0.05–10 µM range. Therefore, in subsequent preparations of the aptasensor 0.05 mM glutaraldehyde and 0.05 µM aptamer were used.

### 3.4. Test for Non-Specific Adsorption

**Figure 5:**
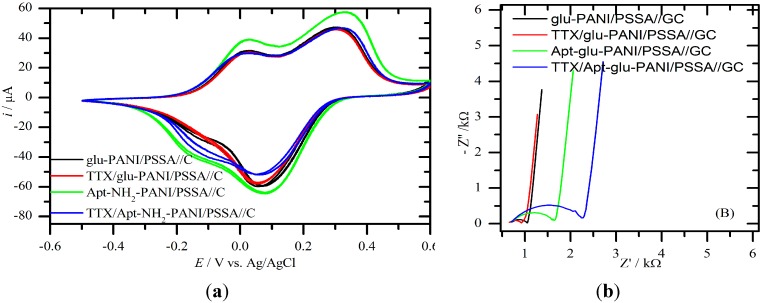
(**a**) CVs and (**b**) EIS spectra of TTX aptasensor (Apt-NH_2_-glu-PANI-PSSA//C) and the control sensor (glu-PANI/PSSA//C) before and after 30 min exposure to 5 μM TTX standard solution in NaOAc buffer (0.1 M, pH 4.8).

In Figure 5 the CVs and EIS spectra of TTX aptasensor (Apt-NH_2_-glu-PANI/PSSA//C) are compared and contrasted with those of a control sensor (glu-PANI/PSSA//C), which contained all components except the aptamer. For the control sensor, it can be seen that the CVs and EIS spectra before and after exposure to the analyte (TTX) did not significantly differ. In contrast, the aptasensor exhibited shifts in peak currents, peak potentials and charge transfer resistance (as measured by the diameter of the semicircles in Figure 5b), thereby confirming that the response obtained for TTX originated from its specific interaction with the immobilized aptamer.

### 3.5. Dynamic Linear Range and Limit of Detection

**Figure 6:**
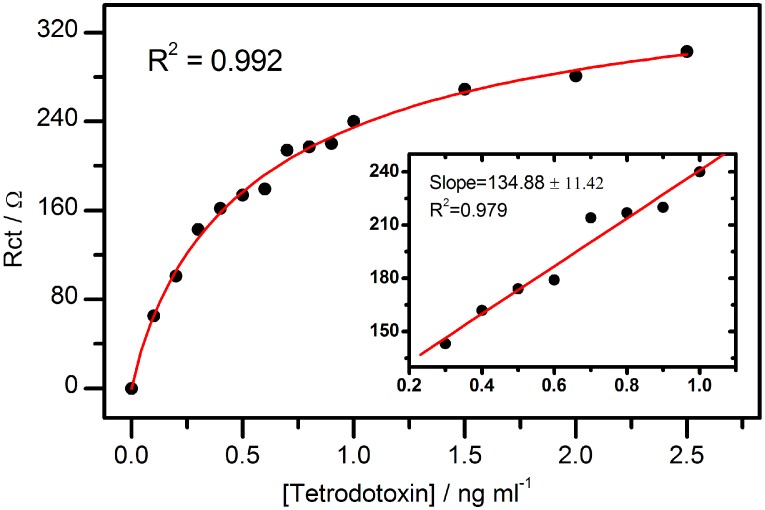
Calibration plot of the impedimetric TTX aptasensor (main graph); and the plot of the analytical linear range of the aptasensor (inset).

The calibration curves of the optimized TTX aptasensor are plotted in Figure 6 for 0–2.5 ng·mL^−1^ TTX and used to determine the sensor’s dynamic linear range (DLR), sensitivity and limit of detection (LOD).

The sensor’s response to TTX revealed an underlying Langmuir adsorption-isotherm process characterized by a hyperbolic increase in *R*_ct_ value with increase in TTX concentration. Therefore, one molecule of the aptamer suggestively binds only one molecule of TTX. The DLR of the TTX aptasensor was estimated to be 0.23–1.07 ng·mL^−1^ TTX (see Figure 6 inset), which is narrower than the values reported for ELISA and SPR methods [5,36,37,38]. The sensitivity and LOD of the aptasensor were calculated to be 134.88 ± 11.42 Ω·mL·ng^−1^ and 0.199 ng·mL^−1^, respectively. The LOD value of the aptasensor is lower than the ones reported by Taylor *et al.* (0.3 ng·mL^−1^) [5] for a surface plasmon resonance (SPR) sensor and Neagu *et al.* (2 ng·mL^−1^, *R*^2^ = 0.924) [3] for an immunosensor.

In Table 1, the analytical figures of merit of the TTX aptasensor (impedimetric aptasensor) are compared with those of other methods reported for TTX analysis. It can be seen that none of the previous studies used electrochemical method to analyze TTX. Though the aptasensor exhibited narrower DLR compared to other techniques, its LOD is comparable to those of LC-MS/MS, SPE-GC/MS, ELISA, SPR and HPLC.

**Table 1:** The analytical parameters of tetrodotoxin determination techniques.

Method	DLR (ng·mL^−1^)	LOD (ng·mL^−1^)	Reference
LC-MS	94–9375	15.6	[39]
LC-MS/MS	1–10	0.1	[40]
HPLC	30–600	1.0	[41]
SPEGC/MS	0.5–10	0.1	[42]
SPR	0.01–10	0.3	[5]
ELISA	2–50	1.0	[43]
	5–500	0.1	[44]
	40–8000	40	[45]
Impedimetric aptasensor	0.23–1.07	0.19	This work

## 4. Conclusions

This is the first reported development of an impedimetric tetrodotoxin aptasensor, in particular and electrochemical TTX sensor in general. The sensor consists of an amine-terminated aptamer cross-linked (with glutaraldehyde) to a p-doped sulfonated polyaniline on glassy carbon electrode. The aptasensor’s LOD value is comparable to both chromatographic and ELISA methods [40,42,44]; and the lower value of the aptasensor’s DLR (*i.e.*, 0.1 ng·mL^−1^) is similar to that of the highly sensitive GC/MS method [42]. This means that for future studies, the sensor can be further developed for application in real samples.
